# Genome wide scan for somatic cell counts in holstein bulls

**DOI:** 10.1186/1753-6561-5-S4-S17

**Published:** 2011-06-03

**Authors:** Giulietta Minozzi, Ezequiel L Nicolazzi, Francesco Strozzi, Alessandra Stella, Riccardo Negrini, Paolo Ajmone-Marsan, John L Williams

**Affiliations:** 1 Parco Tecnologico Padano, Via Einstein, Polo Universitario, Lodi 26900, Italy; 2 Istituto di Zootecnica, Facoltà di Agraria, Università Cattolica del Sacro Cuore, Piacenza, 29122 Italy; 3 IBBA, CNR, Lodi, 26900, Italy

## Abstract

**Background:**

Mastitis is the most costly disease for dairy production, and control of the disease is often difficult, due to its multi-factorial nature. Susceptibility to mastitis is under partial genetic control and the industry uses indirect selection for decreased concentrations of somatic cells in milk to reduce mastitis.

**Methods:**

A genome-wide scan was performed to identify genomic regions associated with deregressed estimated breeding values (EBVs) for somatic cell counts (SCC) in Holstein bulls. In total 1183 proven bulls of the Italian of Holstein population, were genotyped with the BovineSNP50 BeadChip (Illumina, San Diego, CA) and a whole genome association analysis was performed using the R package GenABEL.

**Results:**

Two chromosomal regions showed association with SCC, a region on chromosome 14 with high significance (P < 5x10^-6^) and a region on chromosome 6 with moderate significance (P < 5x10^-5^).

**Conclusions:**

Two regions with effects on SCC have been identified with good statistical support. A further study of these candidate regions will be performed to verify the results and identify the causal mutations.

## Background

Mastitis, an inflammation of the mammary gland caused by an infection with a range of bacteria, is the most costly disease for dairy production. Control of mastitis is difficult due to its multi-factorial nature. Susceptibility to mastitis is under partial genetic control and the industry uses selection on a correlated trait (somatic cells score in milk), to reduce mastitis incidence in the population. Over the last few years, several studies have identified genetic loci putatively associated with somatic cell counts or clinical mastitis [1,2]. The availability of the bovine genome sequence and high density genotyping panels of single nucleotide polymorphisms has allowed a considerable number of bulls worldwide to be genotyped for genomic evaluation and selection. Furthermore, this information can be used to perform association studies with high precision at genome-wide level. The work reported here used genotypic data from the genomic selection project to perform a genome-wide scan with the objective of identifying genomic regions associated to deregressed estimated breeding values (DR-EBVs) for somatic cell counts (SCC) in Holstein bulls.

## Methods

### Animals

The bulls chosen for the genome wide association study were selected from among the 3155 animals progeny tested in Italy with DNA samples available. All these bulls will be used by the Italian National breeders association of Holstein Frisian Cattle (ANAFI) to perform national genomic evaluations.

Selection criteria used for association studies were intended to obtain: i) bulls with high selection index reliability (PFT > 0.75%); and ii) as low relationships between animals in the dataset as possible by trying to keep as many families (father – son couples) as possible. Among the 3155 bulls with biological material available 2109 bulls had appropriate criteria to be included in the study, 1183 of which had been already genotyped with the Bovine 50K SNP chip (Illumina Inc, San Diego).

### Phenotype: deregressed EBV for SCC

The EBV for SCC had a mean of 98.73 ± 5.3 for the 2109 bulls, and a mean 98.77 ± 6.3 in the cohort of 1183 animals included in the study. Furthermore, deregressed EBVs (DR-EBVs) had mean of 0 and a standard deviation of 5. The DR-EBVs and reliabilities for somatic cell counts were derived from a reduced animal model for single records on a single trait. The strategy used to estimate the deregressed estimates was a simplified version of the algorithm of Jamrozik et al. [3], appropriate to a single trait reduced animal model.

### Statistical analysis

Genome-wide association analysis was performed with the GenABEL package in R using a three step GRAMMAR-CG approach, (Genome wide Association using Mixed Model and Regression - Genomic Control) [4,5]. Uncorrected p-values of P < 5 x 10^-7^ were accepted to represent very strong proof of genome-wide association, while p-values between 5 x 10^-7^ and 5 x 10^-5^ were considered as moderately significant associations.

### Genotyping and quality control filters

A total of 1183 progeny tested bulls were genotyped with the BovineSNP50 BeadChip (Illumina, San Diego, CA). Genotype quality assurance was performed within the R statistical environment using the GenABEL package (“check.marker” function) [6]. SNPs were checked for marker call rate (>5%) and minor allele frequency (<5%): markers missing 5% of data and with MAF of less than 5% were removed. Genotyping efficiency of samples was also verified, thus, samples with more than 5% missing data were removed. Classical Multi Dimension Scaling (MDS) was used to explore population substructure and to verify the genetic homogeneity of the dataset prior to analysis.

## Results and discussion

### Quality control

Following quality control checks, 641 markers were excluded because of low call rate and 11404 markers were excluded because of low minor allele frequency. Furthermore, markers on the sex chromosomes were removed from the analysis. A total of 8 samples were removed because of low call rate and other 2 were eliminated because of high autosomal heterozygosity (FDR < 1%). Mean heterozygosity of the dataset after quality check was 0.33 ± 0.01, while the samples removed had heterozygosity higher than 0.63, indicating possible sample contamination. No samples were removed due to high IBS (Identity By State). Mean IBS was 0.70 ± 0.01, based on 2000 autosomal markers, while the threshold for IBS was set to > 0.95. No outliers were identified by Classical Multi Dimension Scaling (MDS).

After quality controls, the final dataset used in the following association analysis contained 1173 samples and 41209 Genome wide SNPs.

### Association analysis

Two chromosomal regions showed associations with SCC, a region on chromosome 14 with high significance (P < 5x10^-6^) and a region on chromosome 6 with moderate significance (P < 5x10^-5^). These two chromosome regions should be further tested to confirm these associations and to potentially identify the causative variations that affect this trait.

A recent review of QTL reported on chromosome 14 [7] identified 10 QTL for disease traits as mastitis, seven of which were related to somatic cell score [8-11]. Interestingly the SNP identified in this study and located on chromosome 14, is actually within 1Mb from the QTL identified by Kaupe et al. [7] to be associated to somatic cell score, but significantly distant from all other chromosomal regions that harbor QTL for clinical mastitis [7].

Furthermore Nilsen et al, [2] characterized a region of chromosome 6 in which QTL for clinical mastitis had been identified, and found the Mucin 7 gene to be significantly associated with the trait. Mucin7 is located close to the casein cluster on chromosome 6. However, the SNP located on chromosome 6 obtained in this study is more than 2 Mb distant from the casein cluster, indicating that different genes could be involved. To confirm the results found in the current study, both SNP identified will be tested in a second independent set of animals.

## Competing interests

The authors declare that they have no competing interests.

## Authors' contributions

GM and JLW wrote the manuscript. JLW and PAM conceived the study. ELN extracted the phenotypic and pedigree information and defined the selection criteria. RN coordinated the sample collection and DNA preparation. GM performed statistical analysis. AS and FS contributed to the data analysis. All authors revised the paper, and approved the final manuscript.

## References

[B1] Klungland H Sabry A Heringstad B Olsen HG Gomez-Raya L Våge DI Olsaker I Ødegård J Klemetsdal G Schulman N Vilkki J Ruane J Aasland M Rønningen K Lien S Quantitative trait loci affecting clinical mastitis and somatic cell count in dairy cattle Mamm Genome 2001 12 837 42 10.1007/s00335001-2081-3 11845286

[B2] Nilsen H Olsen HG Hayes B Nome T Sehested E Svendsen M Meuwissen TH Lien S Characterization of a QTL region affecting clinical mastitis and protein yield on BTA6 Anim Genet 2009 40 701 12 10.1111/j.1365-2052.2009.01908.x 19466933

[B3] Jamrozik J Schaeffer LR Jansen GB Approximate accuracies of prediction from random regression models Livest 2000 66 85 92 10.1016/S0301-6226(00)00158-5

[B4] Amin N van Duijn CM Aulchenko YS A genomic background based method for association analysis in related individuals PLoS ONE 2007 2 12 e1274 10.1371/journal.pone.0001274 18060068 PMC2093991

[B5] Aulchenko YS de Koning DJ Haley C Genomewide rapid association using mixed model and regression: a fast and simple method for genome-wide pedigree-based quantitative trait loci association analysis Genetics 2007 177 577 85 10.1534/genetics.107.075614 17660554 PMC2013682

[B6] Aulchenko YS Ripke S Isaacs A van Duijn CM GenABEL: an R package for genome-wide association analysis Bioinformatics 2007 23 1294 6 10.1093/bioinformatics/btm108 17384015

[B7] Wibowo TA Gaskins CT Newberry RC Thorgaard GH Michal JJ Jiang Z Genome assembly anchored QTL map of bovine chromosome 14 Int J Biol Sci 2008 4 406 14 10.3923/ijb.2008.406.420 19043607 PMC2586679

[B8] Ashwell MS Da Y VanRaden PM Rexroad CE Jr Miller RH Detection of putative loci affecting conformational type traits in an elite population of United States Holsteins using microsatellite markers J Dairy Sci 1998 81 1120 1125 10.3168/jds.S0022-0302(98)75674-7 9594401

[B9] Kaupe B Brandt H Prinzenberg EM Erhardt G Joint analysis of the influence of CYP11B1 and DGAT1 genetic variation on milk production, somatic cell score, conformation, reproduction, and productive lifespan in German Holstein cattle J Anim Sci 2007 85 11 21 10.2527/jas.2005-753 17179535

[B10] Rodriguez-Zas SL Southey BR Heyen DW Lewin HA Interval and composite interval mapping of somatic cell score, yield, and components of milk in dairy cattle J Dairy Sci 2002 85 3081 91 10.3168/jds.S0022-0302(02)74395-6 12487475

[B11] Rupp R Lagriffoul G Astruc JM Barillet F Genetic parameters for milk somatic cell scores and relationships with production traits in French Lacaune dairy sheep J Dairy Sci 2003 86 1476 1481 10.3168/jds.S0022-0302(03)73732-1 12741573

